# Periodontal inflammation recruits distant metastatic breast cancer cells by increasing myeloid-derived suppressor cells

**DOI:** 10.1038/s41388-019-1084-z

**Published:** 2019-11-04

**Authors:** Ran Cheng, Sandrine Billet, Chuanxia Liu, Subhash Haldar, Diptiman Choudhury, Manisha Tripathi, Monirath Hav, Akil Merchant, Tao Hu, Haiyun Huang, Hongmei Zhou, Neil A. Bhowmick

**Affiliations:** 1 State Key Laboratory of Oral Diseases, West China Hospital of Stomatology, Sichuan University, Chengdu, Sichuan China; 2 Department of Medicine, Cedars-Sinai Medical Center, Los Angeles, CA USA; 3 Department of Preventive Dentistry, West China Hospital of Stomatology, Sichuan University, Chengdu, Sichuan China; 4 Department of Periodontology, West China Hospital of Stomatology, Sichuan University, Chengdu, Sichuan China; 5 Department of Research, Greater Los Angeles Veterans Administration, Los Angeles, CA USA

**Keywords:** Cancer microenvironment, Metastasis

## Abstract

Periodontal diseases can lead to chronic inflammation affecting the integrity of the tooth supporting tissues. Recently, a striking association has been made between periodontal diseases and primary cancers in the absence of a mechanistic understanding. Here we address the effect of periodontal inflammation (PI) on tumor progression, metastasis, and possible underlining mechanisms. We show that an experimental model of PI in mice can promote lymph node (LN) micrometastasis, as well as head and neck metastasis of 4T1 breast cancer cells, both in early and late stages of cancer progression. The cervical LNs had a greater tumor burden and infiltration of MDSC and M2 macrophages compared with LNs at other sites. Pyroptosis and the resultant IL-1β production were detected in patients with PI, mirrored in mouse models. Anakinra, IL-1 receptor antagonist, limited metastasis, and MDSC recruitment at early stages of tumor progression, but failed to reverse established metastatic tumors. PI and the resulting production of IL-1β was found to promote CCL5, CXCL12, CCL2, and CXCL5 expression. These chemokines recruit MDSC and macrophages, finally enabling the generation of a premetastatic niche in the inflammatory site. These findings support the idea that periodontal inflammation promotes metastasis of breast cancer by recruiting MDSC in part by pyroptosis-induced IL-1β generation and downstream CCL2, CCL5, and CXCL5 signaling in the early steps of metastasis. These studies define the role for IL-1β in the metastatic progression of breast cancer and highlight the need to control PI, a pervasive inflammatory condition in older patients.

## Introduction

Periodontal diseases are characterized by inflammation affecting the integrity of the tooth supporting tissues, among them gingivitis and periodontitis are the most common. Periodontitis affects nearly half of the US adult population [1]. Strikingly, the incidence of periodontitis afflicts 70% of the elderly over 65-population. Besides tooth loss, periodontitis can also increase the patient’s risk for multiple diseases, among them: atherosclerosis, adverse pregnancy outcomes, rheumatoid arthritis, pneumonia, and cancer [2]. The most relevant carcinogenic link between periodontitis and cancer is oral squamous cell carcinoma [3–7]. Periodontal pathogens such as *Porphyromonas gingivalis* (*P. gingivalis*), *Fusobacterium nucleatum* (*F. nucleatum*), and *Prevotella intermedia* can potentially serve to initiate or promote tumor development, as seen for gastric cancer with *Helicobacter pylori* infection [8]. Interestingly, a striking association has also been demonstrated between periodontal diseases and nonhead and neck cancers [9–11]. High levels of *P. gingivalis* and *F. nucleatum* have been attributed to pancreatic and colorectal cancer in lymph node (LN) metastases [12, 13]. The mechanism could implicate a direct toxic effect of bacterium and their products, or indirect consequences of inflammation. Neoplastic risk increases when cells have continuous proliferation in an inflammatory environment rich in immune cells, growth factors, activated stroma, and DNA-damage-promoting agents [14]. Periodontitis supports such an inflammatory microenvironment.

Several animal models have been used to mimic cellular complexities that occur in human periodontal diseases. Gram-negative bacteria are thought to be important periodontal pathogens. The lipopolysaccharide (LPS) component of the cell wall of these microorganisms, is a significant inflammatory stimulus that triggers an innate immune response. Thus, injections of LPS into the mouse gingival tissues is capable of recreating a sterile model of periodontal inflammation (PI), not involving the pervasive nature of untreated bacterial infections [15, 16]. Toll-like receptor (TLR) 2 and TLR4 are involved in the recognition of various bacterial cell wall components, including LPS, by potentiating the inflammasome signaling cascade via NF-κB activation, expression of proinflammatory cytokines, and reactive oxygen species (ROS) production [17, 18]. The assembly of the inflammasome protein complex can trigger the maturation and secretion of interleukin-1 beta (IL-1β), induction of downstream cytokines, and pyroptosis, potentiating the inflammatory microenvironment [19–24]. IL-1β, a recognized product of pyroptotic cell death and key cytokine in cancer progression, has already been selected as a target for cancer immunotherapy. However, the efficacy of such antagonists is complicated by the fact that IL-1β is associated with the recruitment of immune cell that can be both pro- and antitumorigenic [25]. Myeloid derived immune suppressor cells (MDSCs) represent a heterogeneous population of immature myeloid cells that include granulocytes, macrophages, and dendritic cells that have protumorigenic properties and are associated with establishing the premetastatic niche [26]. MDSC are correlated with metastases of nonsmall cell lung cancer, breast cancer, and melanoma [27–29]. The macrophage can also contribute to the premetastatic niche, as their recruitment is part of the innate immune surveillance [30]. In this study, we use breast cancer models to study the effect of PI on tumor progression and metastasis.

## Results

### Periodontal inflammation can promote lymph node micrometastasis

**Fig. 1:**
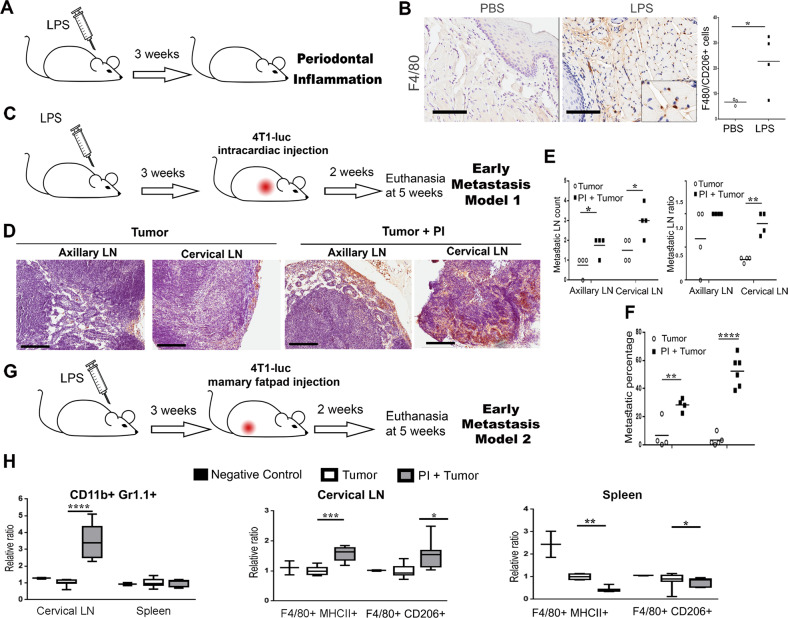
Periodontal inflammation promotes micrometastasis and shows enhanced MDSCs in cervical lymph nodes. **a** Timeline of the PI model, LPS was injected directly in the gingiva of the mice for 3 weeks. **b** Gingiva from PBS and LPS treated mice were stained by F4/80 and FACS quantification of M2 macrophages (F4/8+/CD206+) was assessed (*n* = 3; Bar represents 100 μm). **c** Timeline of the early metastasis model 1 established by intracardiac injection of 4T1 cells. **d** In early metastasis model 1, the axillary and cervical lymph nodes (LNs) were collected for luciferase staining by IHC (Bar represents 200 μm). **e** The numbers of visible LN count (left), as well as the ratio of luciferase-positive LN to total LN were shown (right; *n* = 4). **f** The percentage of metastasis was determined by measuring the ratio of metastasis area to total lymph node area per histologic field. (*n* ≥ 4; LNs lymph nodes; student’s *t* test). **g** Timeline of the early metastasis model 2, mice were injected in the mammary fat pad with 4T1 cells and animals were euthanized at 5 weeks. **h** MDSC and macrophages were measured by FACS in the LN and in spleen. The level of MDSC and macrophages in tumor group were set as 1, then other groups were counted as relative ratio to the tumor group. MDSC and M2 macrophages were significantly elevated in the cervical LN of PI + tumor animals (*n* ≥ 7; LNs lymph nodes; student’s *t* test). (*P < 0.05; ****P < 0.0001)

Inflammatory pathways are key mediators for cancer development and metastatic progression. Using the PI model of chronic LPS injection in the gingiva we observed an immune response after 3 weeks (Fig. 1a, b). As expected, macrophage recruitment was immune-localized to the gingiva and distinguished to be more specifically M2-like macrophage at the inflammatory site, based on the co-expression of F4/80 and CD206 compared with control, PBS injected gum tissue (Fig. 1b). To determine if this inflammatory microenvironment could affect breast cancer metastasis, initially, luciferase-expressing 4T1 breast cancer cells were introduced by intracardiac injection in control and PI mouse models (Fig. 1c). After 2 weeks animals were evaluated for metastatic progression. At this early stage, the mice with PI were found to have developed micrometastasis in lymph nodes (LNs) at a greater frequency compared with the sham inject mice (Fig. 1d, e). Interestingly, there was a greater tumor burden in the cervical LNs compared with the axillary site, based on luciferase localization and histochemical validation of the mice with PI (Fig. 1e, f). The ratio of positive LNs to total LNs was significantly elevated in the mice with PI versus control mice that had sham injected gingiva (Fig. 1e). Of note, similar injection of LPS in the peritoneal cavity (to mimic a more systemic inflammatory state) did not mediate a significant metastatic response of breast tumors (data not shown). Hence, the sterile model of PI seemed to have the potential to not only induce metastasis, but recruit metastasis toward the site of the inflammation.

### Periodontal inflammation enhanced immune infiltrates in cervical lymph nodes in an orthotopic syngeneic model

To better characterize the tumor tropism mediated by PI, a syngeneic metastatic model was tested using orthotopically grafted 4T1 cells in the fourth mammary gland (Fig. 1g). LN micrometastases found in this model were comparable to the cardiac injection metastasis model, in terms of the location of tumor-positive nodes. Here, we focused on the immune infiltrates of the metastatic site and the spleen by FACS analysis. The levels of MDSC and macrophages were markedly elevated in the cervical LNs (Fig. 1h). MDSC recruitment to the LNs of tumor bearing mice with PI were more than threefold greater than the tumor bearing mice without PI (*P* < 0.0001). Previous studies have highlighted the recruitment and expansion of MDSC in the periodontal inflammatory process induced by *P. gingivalis* infection [31]. Likewise, we identified polarized M2 macrophage in the metastatic cervical LNs, characterized by F4/80^+^ and CD206^+^ expression in the tumor bearing mice with PI, compared with sham injected mice allografted with tumor alone (*P* < 0.05). In addition, M1 macrophages, characterized by F4/80^+^ and MHCII^+^ staining, were similarly increased in mice with PI. The presence of MDSC and M2 macrophage in the cervical LNs was suggestive of a tumorigenic niche. Not entirely surprising, the M1 and M2 macrophages in the spleen seemed to be depleted in tumor bearing mice with PI, compared with mice with either tumors or PI alone, in support of an active recruitment process to the site of inflammation. We observed recruitment of macrophage and MDSC to the cervical LNs of mice with PI, concomitant with tumor cells.

### Periodontal inflammation leads to pyroptosis and IL-1β expression in vitro

**Fig. 2:**
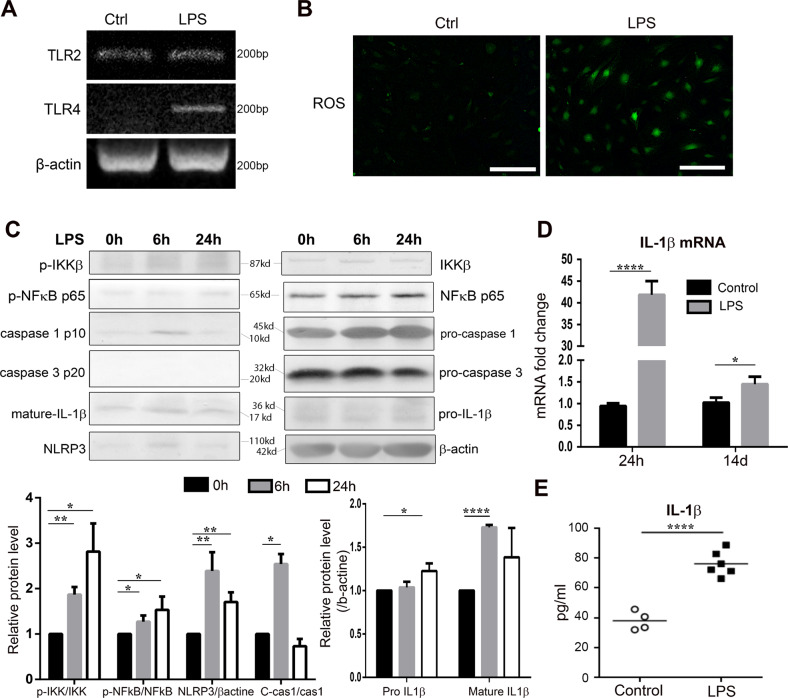
LPS leads to pyroptosis and IL-1β expression of gingival fibroblasts in vitro. **a** Gingival fibroblasts were treated with LPS (1 μg/ml) for 24 h. The mRNA level of TLR2 and TLR4 were measured by RT-PCR. **b** Reactive oxygen species (ROS) were determined by DCFH-DA staining (bar = 100 µm). **c** Gingival fibroblasts were treated with LPS for 6 and 24 h. Protein expressions of IKKβ (phosphorylated and total), p65 NF-κB (phosphorylated and total), NLRP3, caspase 1 (pro- and cleaved p10), caspase 3 (pro- and cleaved p20), and IL-1β were measured by western blot. Cleaved p10 caspase-1 is depicted as C-cas1. Band intensities were assessed using Quantity One. Data (*n* = 3) are shown as means ± SEM. **d** Gingival fibroblasts were treated with LPS (1 μg/ml) for 24 h and 14 d. IL-1β mRNA level was measured by real-time PCR. (*n* = 3) (E) Gingival fibroblasts were treated with LPS for 24 h, exogenously secreted IL-1β was measured by ELISA. (*n* ≥ 4; student’s *t* test, *P < 0.05; **P < 0.01; ****P < 0.0001)

Numerous factors can induce the recruitment and differentiation of MDSCs. The chronic injection of LPS into the gingival tissue, used to initiate the sterile PI model, could mediate a host response by the production of proinflammatory factors capable of recruiting macrophages and MDSC [32]. In trying to characterize the mechanism underlying the metastatic models, we treated primary cultures of mouse gingival fibroblasts with LPS. TLR2 and TLR4 mRNA was found to be elevated by 24 h of LPS treatment (Fig. 2a). Moreover, LPS elevated ROS in the gingival fibroblasts, as localized by 2′,7′-dichlorofluorescin diacetate (DCF-DA) fluorescent staining (Fig. 2b), suggesting the presence of a trigger for nod-like receptor (NLR) inflammasome formation [33]. To explore this pathway, the major components of the pyroptosis cascade were examined by quantitative rtPCR and western blot following LPS incubation, up to 14 days. We found the initiation of signal I, associated with NF-κB activation (phosphorylated p65) occurred within 6 h of LPS stimulation (Fig. 2c). The activation of signal I was further confirmed by IL-1β mRNA measured from LPS stimulated gingival fibroblasts (Fig. 2d). The signal II mediator, NLRP3, was also upregulated within 6 h of LPS stimulation, as were the downstream cleavage of pro-IL-1β by activated caspase 1 in the gingival fibroblasts. Extended exposure to LPS maintained an active state of the pyroptosis pathway. Mature IL-1β intracellular expression was found to be upregulated after longer exposure to LPS. The LPS-induced elevated secretion of IL-1β was measured in the conditioned media by ELISA to confirm signal II activity (Fig. 2e). Inflammasome activation in gingival fibroblasts in the PI models could promote a metastatic immune microenvironment in the gingiva.

**Fig. 3:**
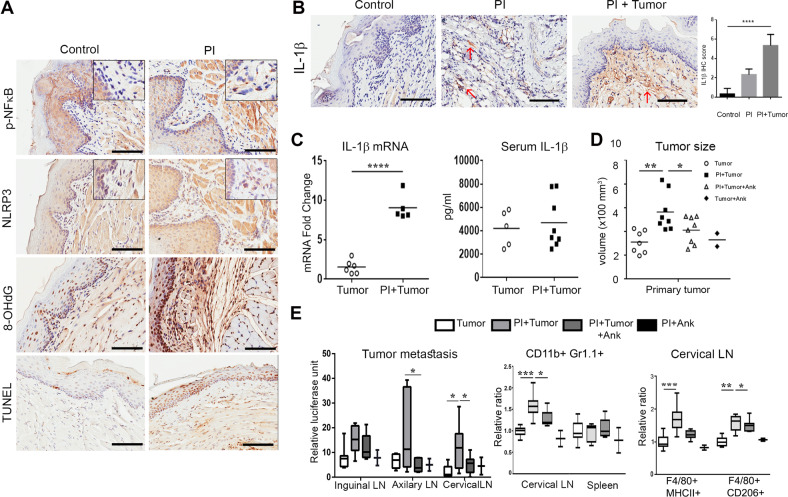
Pyroptosis/IL-1β pathway is enhanced in vivo and can be modulated by anakinra. **a** In the PI model, phosphorylated-p65 NF-κB, NLRP3, 8-OHdG, IL-1β, and TUNEL were detected by IHC (Bar, 100 μm). **b** IL-1β was immuno-localized in gum tissues of mice with PI alone and with orthotopic mammary tumor introduction (Bar, 100 μm). **c** The mRNA expression of IL-1β was measured in the gingiva of the indicated groups. Serum IL-1β was detected by ELISA (*n* ≥ 5; Student’s *t* test). **d** The size of primary tumors was measured (*n* ≥ 7). **e** Early metastasis in lymph nodes were measured by luciferase assay and normalized by protein concentration, where anakinra reduced the metastasis to the axillary and cervical lymph nodes. MDSC content was also decreased by anakinra in cervical lymph node. (*n* ≥ 7; Ank anakinra; ANOVA, *P < 0.05; **P < 0.01; ***P < 0.001; ****P < 0.0001)

We next sought to determine the relevance of IL-1β expression by gingival fibroblasts on breast cancer metastatic progression. We found that PI tissues had expression of NF-κB phosphorylated-p65 and NLRP3 in the fibroblastic cells, indicating active inflammasome signal I and signal II signaling (Fig. 3a and Supplementary Fig. S1). Compared with sham injected mice, ROS and cell death were enhanced in the PI afflicted mouse gingival tissues, as observed by 8-OHdG and TUNEL staining, respectively. An output of inflammasome signaling, IL-1β, was significantly upregulated in gingival stroma by immune-localization and confirmed by quantitative-PCR (Fig. 3b, c). However, since systemic IL-1β measured in the serum was not appreciably altered, it suggested a more localized periodontal signaling in the tumor bearing mice with PI. We chose to block IL-1β to test the role of pyroptosis on immune infiltration and metastatic tumor progression. Accordingly, anakinra, an IL-1 receptor antagonist, was administered to tumor bearing mice with PI (Fig. 3d). In this model, anakinra was found to inhibit distal metastasis, both in axillary and cervical LNs concurrent with diminished MDSC and M2 macrophage recruitment, similar to the level of control animals, but no significant impact on M1 macrophages (Fig. 3e and Supplementary Fig. S2).

**Fig. 4:**
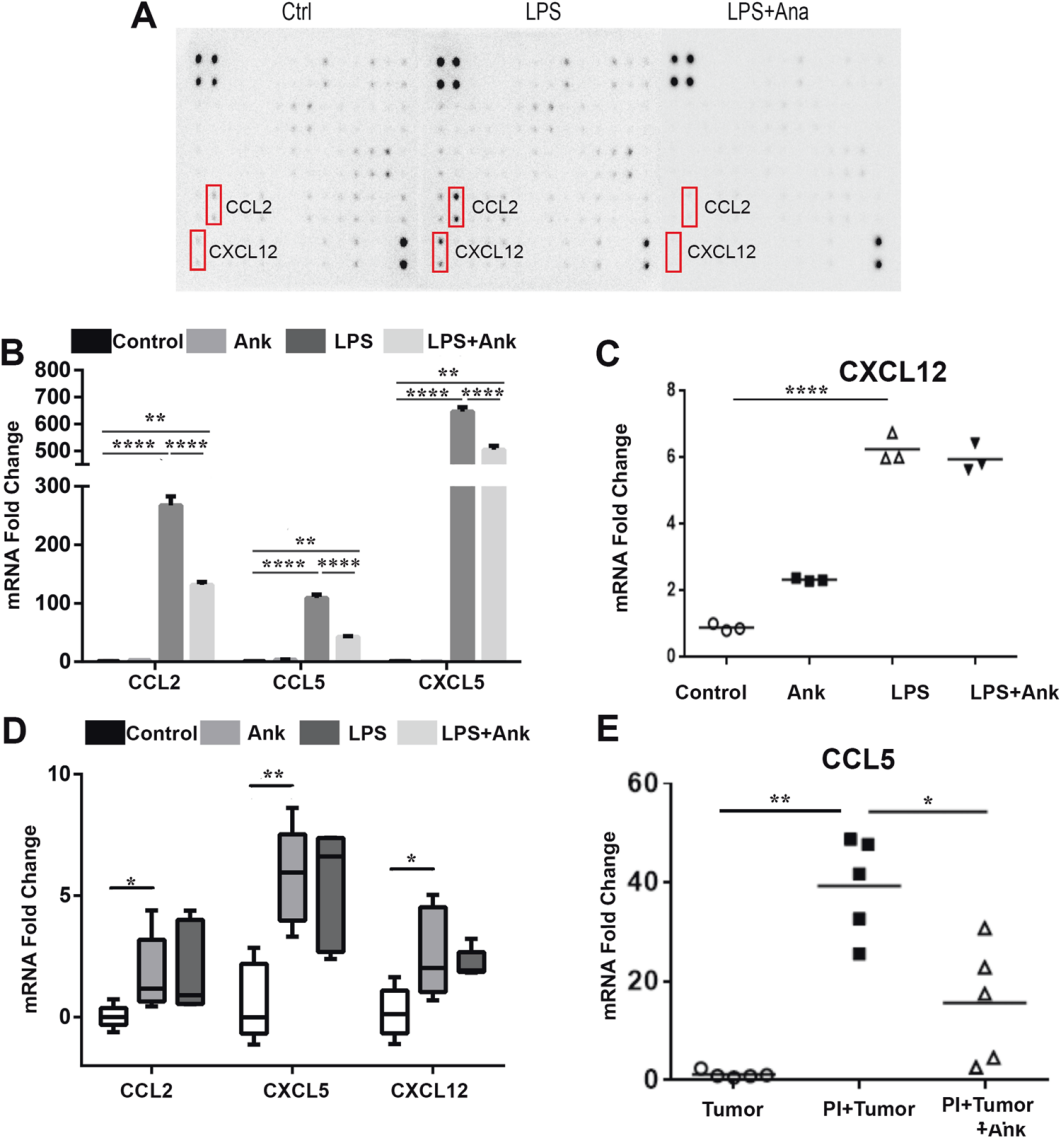
LPS increases expressions of inflammatory genes and/or chemoattractants. Anakinra partly diminishes the effects of LPS. **a** Gingival fibroblasts were treated with LPS for 24 h, with or without anakinra in vitro. Cytokine assay showed anakinra inhibited the expressions of CXCL12 and CCL2, which were increased by LPS. **b**, **c** mRNA level of CXCL5, CCL2, CCL5, and CXCL12 were measured in vitro. LPS enhanced all the chemokines and anakinra inhibited them except for the mRNA level of CXCL12 (*n* = 3; ANOVA or student’s *t* test). **d**, **e** mRNA level of CXCL5, CCL2, CCL5, and CXCL12 in the gingival tissue were measured in vivo in the 3-animal group. (*n* = 5; Ank anakinra, ANOVA or student’s *t* test, *P < 0.05; **P < 0.01; ****P < 0.0001)

To evaluate the role of IL-1β in immune cell recruitment, a panel of chemokines and cytokines were measured from gingival fibroblasts treated with LPS with or without anakinra. LPS was found to elevate CCL2 expression by almost sixfold and CXCL12 expression by threefold (Fig. 4a). Anakinra inhibited the induction of both these cytokines promoted by LPS. CXCL12 and CCL2 have been implicated in MDSC generation, function, and recruitment [34]. The regulation of CCL2 by IL-1β was corroborated at the level of mRNA expression (Fig. 4b). Of interest, other MDSC regulatory cytokines, CCL2, CCL5, and CXCL5, were also found to be upregulated by LPS in an IL-1β-dependent manner, based on the capacity of their inhibition by anakinra (Fig. 4b). CXCL12 expression was significantly elevated by LPS, independent of IL-1ß, based on the lack of effect by anakinra (Fig. 4c). CCL2, CXCL12, CCL5, and CXCL5 mRNA was significantly upregulated in PI gingival tissues compared with control gingival tissues in mice (Fig. 4d, e). However, only the mRNA expression of CCL5 induced in the PI tissues was significantly suppressed by anakinra, in validating its role as an IL-1β downstream target with the capacity to recruit MDSC and macrophages.

### Periodontal inflammation can promote breast cancer metastasis to the head and neck site

**Fig. 5:**
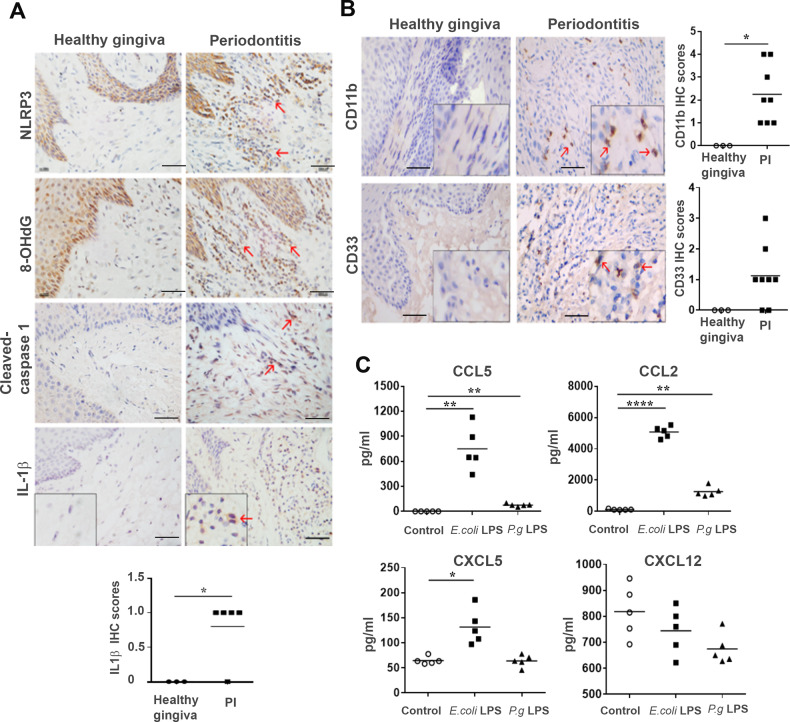
Human gingiva from periodontitis patients show activation of MDSC and express inflammatory chemokines. **a** NLRP3, 8-OHdG, cleaved-caspase 1, and IL-1β were detected in periodontitis specimens by IHC. (Bar represents 50 μm) **b** human CD33+ and CD11b+ MDSC subsets were detected by IHC (three healthy gingiva and 5–8 periodontitis specimens). The number of CD33+ or CD11b+ cells were increased in periodontitis (student’s *t* test; Bar represents 50 μm) **c** human gingival fibroblasts were cultured in vitro and were stimulated by *E.coli* LPS or *P. gingivalis* LPS for 24 h. CCL2, CCL5, CXCL5, and CXCL12 in cell supernatant were measured by ELISA. *E.coli* LPS and *P. gingivalis* LPS highly increased the secretion of CCL2, CCL5 compared with the control. The secretion of CXCL5 was merely increased by *E.coli* LPS. (*n* = 5; student’s *t* test, *P < 0.05; **P < 0.01; ****P < 0.0001)

The PI mouse models produced a histopathological milieu similar to that observed in human periodontitis, characterized by infiltration of leukocytes, high levels of proinflammatory cytokines, collagen degradation, and alveolar bone resorption. Therefore, human gingiva samples were evaluated for components of the pyroptosis pathway and downstream cytokine expression. Human gingiva samples were immunohistochemically characterized for the expression of NLRP3, 8OHdG, cleaved-caspase-1, and IL-1β in periodontitis specimens, compared with that in normal gingival tissues (Fig. 5a and Supplementary Fig. S1B). Heterogeneous staining was observed in each periodontitis specimen with an overall elevation for MDSC markers, CD11b^+^ and CD33^+^, compared with the healthy gingiva (Fig. 5b). Of note, while the variance was similar in most groups of the mouse models, the variance in the human periodontitis patient values were significantly greater. When human gingival fibroblasts were stimulated with either *E. coli* LPS or *P. gingivalis* LPS, CCL5, and CCL2 were significantly upregulated compared to control. CXCL5 was also significantly elevated by *E. coli* LPS to a lesser extent, but not by *P. gingivalis* LPS (Fig. 5c). However, CXCL12 levels were not appreciably affected by LPS treatment.

**Fig. 6:**
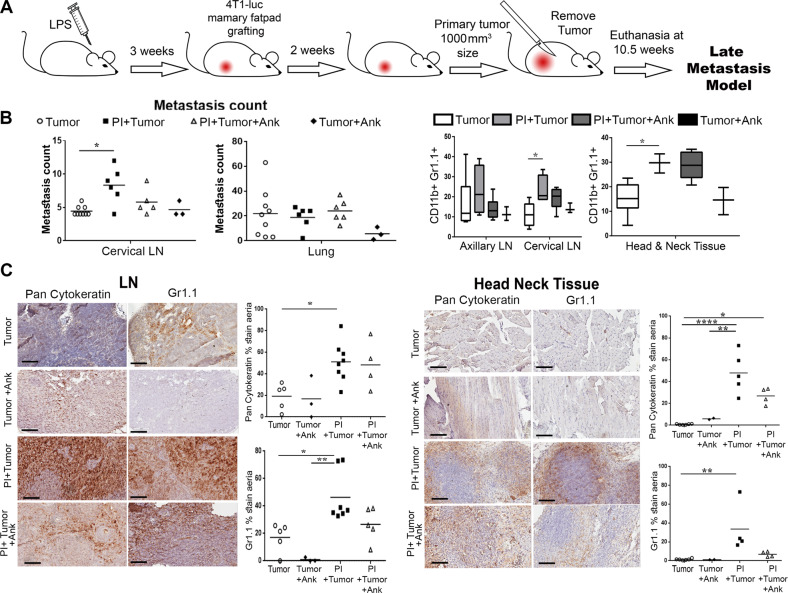
Periodontal inflammation can promote head and neck metastasis. **a** Timeline of the late metastasis model, PI was induced with LPS for 3 weeks then mice were injected in the mammary fat pad with 4T1 cells, the primary tumor was let to grow until it reached 100 mm^3^ in size, surgery was accomplished to extract this tumor and animals were let to grow metastasis and euthanized at 10.5 weeks. **b** Metastasis were observed and counted in lungs and lymph node in the individual animal groups in the presence or absence of anakinra. (*n* ≥ 3; ANOVA). **c** IHC staining of Pan-cytokeratin and Gr1 from the lymph node and the cervical tissue of late metastasis model. (*n* ≥ 3; Ank anakinra, ANOVA, *P < 0.05; **P < 0.01; ****P < 0.0001)

To expand the scope of the findings from the short-term models of LN metastasis, we tested the capacity for the role of localized inflammation to promote homing. The two short-term models used previously supported the capacity of a localized inflammation to generate a localized tumor-supportive niche. Nevertheless, we only observed micrometastasis to LNs in these models. So, we developed a model where we introduced luciferase-expressing 4T1 in the mammary fat pad of mice with PI, and when primary tumors reached 0.8–1 cm^3^ volume they were resected. The mice were sacrificed 10 weeks after initial tumor introduction for evaluation of metastasis development (Fig. 6a). Both control and experimental groups developed lung metastasis independent of PI or anakinra treatment, indicating a localized inflammation had limited systemic effects (Fig. 6b). However, there was a significant elevation of tumor-positive cervical LNs in the mice with PI. Further, metastases were localized in neck and jaw tissues were confirmed histologically with pan-cytokeratin staining, independent of the LNs (Fig. 6c). In this model system, MDSCs were elevated, coincident with tumor cells in the neck LNs. However, anakinra failed to inhibit metastasis and MDSC recruitment in these longer-term studies, supporting the concept that pyroptosis and downstream inflammatory cascade contribute to the early stages of premetastatic niche generation facilitating tumor engraftment. As inhibiting IL-1β had limited efficacy as a therapeutic for metastatic expansion in the longer term, the steps of subsequent tumor expansion seem to be independent of stromal pyroptotic cascade initiated by PI.

**Fig. 7:**
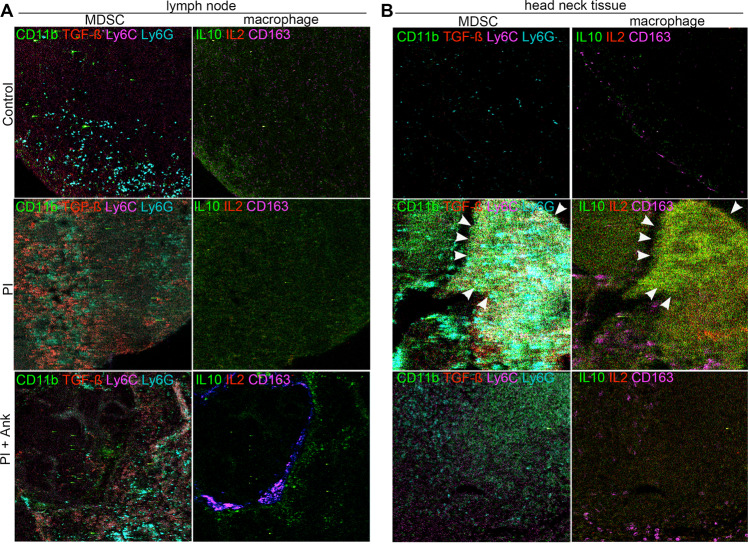
Multiplex protein detection of head and neck and lymph node metastasis. CyTOF based detection of phenotypic and functional markers on paraffin embedded tissue sections of the late stage tumor-bearing mouse control, periodontitis, and periodontitis model administered anakinra. **a** Evaluation of CD11b, Ly6C, Ly6G, TGF-β, CD163, IL-10, and IL-2 expression were detected in lymph nodes from the late breast cancer metastasis model. **b** Detection of these same markers were performed on the neck tissues of the same model. Arrowhead indicates tumor metastasis. The overlaid pseudo-colored images for MDSC and macrophage here are individually illustrated in Supplementary Fig. S3

To further characterize the premetastatic niche and the tumor itself, the different myeloid populations were localized by multiplexed mass cytometry of the tissue sections. The CyTOF panel included a combination of phenotypic (CD11b, Ly6C, Ly6G, and CD163) and functional (IL-10, TGF-β, and IL-2) markers. Granulocytic MDSC (CD11b^+^, Ly6G^+^, Ly6C^low^) recruitment to the LNs in the neck and associated TGF-ß expression seemed to be the greatest in the PI mouse model, compared with either control or the PI mice treated with anakinra (Fig. 7a, Supplementary Fig. S3A). While MDSC recruitment was observed in the control LN of the tumor bearing mice, associated expression of immune suppressive cytokines, IL-10, IL-2, or TGF-ß was not observed. Interestingly, there was greatest CD163^+^ macrophage recruitment to the LNs in the control, tumor bearing mice that had negligible metastatic engraftment to the neck. The LNs of the anakinra treated periodontitis model had both MDSC and macrophage recruitment, with associated TGF-ß and IL-10 expression detectible. Tumor engraftment to the head/neck tissue in the tumor bearing periodontitis model, not surprisingly was associated with greatest level of both MDSC (CD11b^+^, Ly6G^+^, and Ly6C^low^), and to a lesser extent macrophage (Fig. 7b, Supplementary Fig. S3B). The expression of IL-2, IL-10, and TGF-ß was also highest in the neck area of this model. The administration of anakinra to the periodontitis tumor bearing model also had appreciable level of MDSC and some macrophage in the neck area, but the expression of the immune-suppressive cytokines was (IL-2, IL-10, and TGF-ß) was diminished. Multiplexed staining of the limited mouse tissues enabled localization of the MDSC and macrophage with their functional markers, that likely contributed to the resulting metastatic homing of the breast tumors to the head and neck area.

## Discussion

**Fig. 8:**
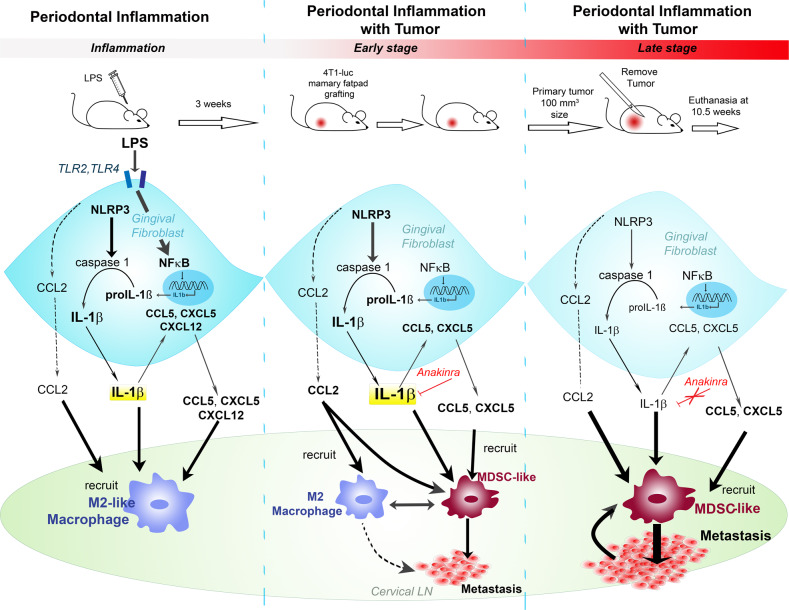
Possible mechanism underling periodontal inflammation-induced metastasis. Bacterial wall component, LPS, induces caspase 1 activation and proptosis pathway signaling in gingival fibroblasts in instances of PI. The proinflammatory cytokine, IL-1β, as well as CCL2, CCL5, and CXCL5 are produced and initiate M2 macrophage. In the early stages of primary breast tumor progression, where there is no expected metastasis, IL-1β and the produced cytokines recruit M2 macrophages, as well as MDSC to the lymph node and head and neck area. In the later stages when metastasis has established, MDSC are the main cells implicated and have a role in increasing the tumor burden in the lymph node and head and neck area, where the role of IL-1β is dispensable

Recently, new links have been established between host–microbe interactions and its related diseases. Examples of this include the role of gut microbiota in Parkinson’s disease [35], diabetes [36], and obesity [37]. The oral microbes can mediate chronic inflammation and affect all stages of tumor development including our finding of metastatic homing. In this present study we provided evidence that PI enhanced tumor metastasis in both early and late stage of cancer progression through an active role of the gingival fibroblasts (Fig. 8). Using models of breast cancer metastasis in the context of PI, we demonstrated the specific role of inflammasome signaling in steps of cancer progression. Chronic LPS administration model of PI mimicked the human disease in terms of immune infiltrates, including polarized macrophage recruitment to the gingiva (Figs. 1, 5). Inflammasome signaling observed in gingival fibroblasts of the sterile PI models, reflected in the human disease, can play a role in the metastatic homing. More specifically, TLR2 and TLR4 activation and IL-1β in mouse gingival fibroblasts mediated CXCL5, CCL2, CCL5, and CXCL12 expression to initiate an acute immune response supporting macrophage and MDSC recruitment. Of note, although human periodontitis was associated with elevated IL-ß expression and MDSC recruitment, LPS appreciably only induced CCL5 and CCL2 in human gingival fibroblasts. The increased expressions of CCL5 were IL-1β dependent in the PI mouse models, as its expression was limited by anakinra. MDSC recruitment and macrophage polarization in the early stages of metastasis seem to be dependent on IL-1β. However, since MDSC can be recruited by a multitude of chemokines including CXCL5, CXCL12, CCL2, and CCL5, limiting CCL5 by IL-1ß antagonism did no appreciably limit MDSC recruitment under chronic administration. Although effective in the early stages of metastatic progression to the LNs, anakinra could not prevent breast cancer metastasis and associated MDSC recruitment in later stages of metastatic progression to the neck tissue.

In this study MDSC recruitment to tumors was associated with PI-induced metastasis. MDSCs were detected at the metastatic sites, including LNs as well as head and neck tissues. CyTOF data demonstrated the clear presence of granulocytic-MDSC (Cd11b^+^, Ly6G^+^, and Ly6C^low^) in the premetastatic niche, as well as in the tumor of the head and neck tissues. This data also confirmed a colocalization of IL-10, IL-2, and TGF-β showing the immunosuppressive expression pattern initiated by these MDSC. While TGF-ß expression is also a feature of M2 macrophage, no such co-expression was observed in our tissues. Activated M2 macrophage are reported to have low IL-2 expression in response to IL-6 [38], interestingly the inhibition of IL-1ß signaling by anakinra likely blocked IL-6 to possibly enable the observed elevation of IL-2 localization with the macrophage. Although both granulocytic and monocytic MDSC have immunosuppressive properties, the granulocytic MDSC are associated with greater expression of ROS and recruitment to the sites of metastasis [39]. Similarly, we identified the gingival fibroblasts exposed to LPS to generate ROS (Fig. 2). Incidentally, *P. gingivalis* is reported to promote the expansion of MDSC and consequently suppressed the host response [31]. However, our finding that 2/8 periodontitis specimens from noncancer patients had elevated CD11b^+^/CD33^+^ MDSC, suggested that its infiltration can be an inherent feature of periodontitis, rather than tumor progression (Fig. 5). As such, in the PI mouse models, MDSC recruitment was a likely precursor to tumor infiltration [40]. The pyroptosis cascade has been detected in inflamed human periodontium, including gingival fibroblast and periodontal ligament cells in our previous studies [15, 41]. IL-1β in saliva or gingival crevicular fluid has even been speculated as a potential biomarker for periodontal disease [42]. The pyroptotic-process induced by PI contributed to the maturation and secretion of IL-1β in mouse gingival fibroblasts. Inhibition of IL-1β by anakinra suppressed tumor metastasis in the head and neck region as well as in LN and even reduced the growth of the mammary primary tumor in the early stages of disease progression. Nevertheless, anakinra failed to be beneficial in the later stages of metastasis where MDSC were involved. Similarly, targeting CCL5, an IL-1ß target, in breast cancer with anti-CCL5 antibodies has been described to decrease the immunosuppressive activity of MDSCs and reduce tumor metastasis [43]. However, as other cytokines, not necessarily a product of inflammasome activity, are left unchecked by IL-1β or CCL5 inhibition, MDSC recruitment and tumor metastasis would be unaffected in the long run.

Inflammasome activity has long been considered essential for host defense against pathogens, implicated in autoimmune diseases, and primarily relegated to a function of immune cells. We have previously demonstrated evidence of inflammasome activity in bladder fibroblastic cells [44, 45]. Herein we observed the inflammasome signaling in the inflamed gingival fibroblasts. Inflammasome activation has gained consideration in the development of cancer as well as its therapy as its can have both protumorigenic or antitumorigenic effects [46]. In lung cancer, inflammasome activity generates IL-1β and IL-8 in promoting EMT and secretion of cytokines that modulate the tumor microenvironment to promote lung cancer progression [47]. In breast cancer, IL-1β production is associated with higher rate of recurrence and is critical for tumor proliferation, angiogenesis, migration, and invasion [24]. Our study demonstrated a direct link between chronic PI and a significant breast cancer metastatic risk. The metastatic niche formed by the gingival fibroblasts was capable of recruiting macrophage, cancer epithelia, and MDSC. Accordingly, inflammasome inhibitors have been proposed to limit tumor progression. The early steps of PI seem to be controlled by IL-1β and macrophages in establishing a metastatic niche, whereas in presence of the tumor and MDSC cells in a later metastatic phase support tumor expansion. However, this study demonstrates such a therapeutic strategy of inflammasome activity would have limited efficacy as a single agent. But, interventions for periodontitis can be considered an effectual preventative for breast cancer metastasis to the head and neck region.

## Materials and methods

### Periodontal inflammation and tumor metastasis mice models

Procedures and animal experiments were approved by Institutional Animal Care & Use Committee (IACUC003638) at Sinai Medical Center. Four-week-old, female BALB/c mice (16–22 g Harlan Laboratories, Indianapolis IN) were used in the syngeneic xenografting and PI models described. The PI model was generated as previously reported by injecting 10 μg LPS (*Escherichia coli* LPS, Sigma-Aldrich, St Louis, MO) in PBS or 10 μl endotoxin-free PBS vehicle into mandibular buccal mucosa 2 times a week for 3 weeks [15, 16]. The early metastasis models were established as follows: 3 weeks following the PI model set, mice received an injection of 4T1cells (5 × 10^4^ cells) intracardially (early metastasis model 1) or received 4T1 cells (10^5^ cells) in the mammary fat pad (early metastasis model 2). Sample size of at least five mice per group were selected to achieve 80% power. Both experiments were terminated 2 weeks following cancer cell injections. Tumor size was measured by Vernier caliper and tumor volumes calculated according to the formula *V* (mm^3^) = *L* (major axis) × *W* (minor axis) × H × 6/π. The axillary, cervical LNs, and gingival tissues were collected for further experiment. The late metastasis model was established after 3 weeks of PI model establishment, where the 4th mammary fat pad was injected with 10^5^ 4T1 cells. The anakinra (20 mg/kg, Amgen Inc, Thousand Oaks, CA) was administered daily, starting on the day of tumor introduction. Following the injection/grafting of tumors the mice were randomized prior to allocating to the control and treatment groups. Tumors were resected when they reached 100 mm^2^ and metastatic progression allowed to progress for up to 10.5 weeks. The axillary, cervical LNs, the neck tissue, and gingival tissue were collected for further evaluation. The investigators were blinded to the group at the time of allocation to the tumor or treatment groups. However, the groups were known to the investigators during data collection and analysis.

### Luciferase assay

LNs were treated with luciferase lysis buffer (0.1 M potassium phosphate buffer, 1 mM DTT, 2 mM EDTA, 1% Triton X-100, and pH 7.8) for 10 min. Luciferase activity was assayed using luciferase substrate buffer (Promega, Madison, WI). Protein concentrations were evaluated using BCA protein assay kit (Thermo Fisher Scientific Inc., Waltham, MA) on a microplate reader (Bio-Rad Laboratories, Hercules, CA). Relative luciferase Unit was calculated on a Luminometer (Monolight™ 3010, BD Biosciences, San Jose, CA) according to the formula, Relative luciferase Unit = (reporter activity − background)/protein concentration.

### Flow cytometry

LNs and spleen tissues were prepared using mechanical dissociation of minced tissue to obtain single cell suspensions. The following antibodies were used for flow cytometry: anti-Ly6G/Ly6C (Cat #58-5931-82), anti-Cd11b (Cat #12-0112-82), anti-CD86 (Cat #11-0862-82), anti-F4/80 (Cat #17-4801-82), anti-MHCII (Cat #13-5321-82), and anti-CD11c (Cat #11-0116-42) all from eBioscience, San Diego, CA) and anti-CD206 (clone MR5D3; BioRad). After being stained with a standard protocol, all events were analyzed with FlowJo software (FlowJo, LLC, Ashland, OR) and frequencies among live cells were obtained.

### Cell culture

Mouse gingival fibroblasts were cultured from mandibular buccal gingival tissues obtained from 6-week-old female Balb/c mice. The gingival tissues were cut into 1 mm^3^ pieces and maintained in Alpha Modifications Minimum Essential Medium (α-MEM) with 10% fetal bovine serum supplemented with 100 U/ml penicillin, and 100 μg/ml streptomycin (all from Cambrex, Walkersville, MD) at 37 °C with 5% CO_2_. Cells between passage 3 and 7 were used.

The 4T1 mouse mammary carcinoma cells were obtained from the American Type Culture Collection (ATCC, Manassas, VA) and were cultured in RPMI 1640 medium supplemented with 10% FBS, 100 U/ml penicillin, and 100 μg/ml streptomycin. Human gingival fibroblasts were cultured following a previously published report [15].

### Cell transfection

4T1-Luc cells were established as follows: the pGL4 luciferase reporter vector was obtained from Promega. This vector containing a hygromycin B selectable marker and was transfected into 4T1 cells by using Nucleofector™ Kit V (Lonza Group Ltd., Basel, Switzerland) with the Amaxa Nucleofector (Lonza Group Ltd.), followed by a 30 μg/ml hygromycin B (Invitrogen, Carlsbad, CA) selection for 7 days. The expression level of luciferase was then evaluated by luciferase assay.

### RT-PCR and Realtime-PCR

Mouse gingival fibroblasts were serum-starved for 24 h, then treated with 1 μg/ml LPS with or without 500 ng/ml anakinra for 24 h. Total mRNA was isolated from gingival fibroblasts or gingival tissue using RNeasy mini kit (Qiagen, Valencia, CA). cDNA was synthesized from 1 μg of total RNA with the iScript cDNA kit (Bio-Rad Laboratories). Receptors mRNA expression was determined by quantitative RT-PCR using primers in Supplementary Table S1 and GoTaq Green Master Mix (VWR International, Radnor, PA). Expression of mRNA was normalized to β-actin levels and expressed as fold change using the comparative threshold cycle (Ct) method (2−ΔΔCt). Primers used were described in Supplementary Table 1.

### Reactive oxygen species (ROS) staining

Gingival fibroblasts were serum-starved for 24 h, then treated with 1 μg/ml LPS for 24 h. Cells were further cultured in the presence of 20 μg MDCF-DA (Sigma-Aldrich) for 20 min and imaged with a fluorescence microscope (Olympus Fsx100, Olympus Corporation, Tokyo, Japan).

### Cytokine assay

Gingival fibroblasts were serum-starved for 24 h, then treated with 1 μg/ml LPS with or without 500 ng/mg anakinra for 24 h. Cytokine concentrations in cell lysates were then determined using the Mouse Cytokine Array C3 (RayBiotech, Inc., GA) following manufacturer’s instructions. Data were quantitated with Quantity One (Bio-Rad Laboratories)

### Western blot

For short-term cultures, gingival fibroblasts were serum-starved for 24 h, then treated with 1 μg/ml LPS for 24 h. For long-term cultures, gingival fibroblasts were treated with 1 μg/ml LPS in α-MEM containing 2% FBS for 14 days. Proteins were detected following a previously published western blot procedure [15]. The following proteins were detected by the following antibodies: anti-caspase 1 (Cat# 14367; Gene Tex Inc., Irvine, CA), anti-caspase 3 (Cat# 611048; BD Transduction Laboratories, San Jose, CA), anti-IL-1β (Cat# AF-201-NA; R&D Systems Inc., Minneapolis, MN), anti-NLRP3 (Cat#LS-C162911; LifeSpan Biosciences, Inc., Seattle, WA), anti-NF-κB p65 (Cat#3033; phosphorylated Ser 536, Cell Signaling Technology, Inc., MA, USA), anti-NF-κB p65 (Cat# sc-71675; Santa Cruz Biotechnology, Inc., Dallas TX), anti-IKKβ (Cat# ab32041; Abcam, MA), anti-IKK beta (Cat# ab59195; phosphorylated Y199, Abcam), β-actin (Cat# sc-69879; Santa Cruz Biotechnology, Inc.).

### CyTOF

Literature was reviewed to identify markers relevant to the tumor and premetastatic niche. Metal conjugated antibodies were obtained with custom conjugations with Fluidim Maxpar Antibody Labeling Kit (Supplementary Table S2). Antibodies were validated on neck area and LNs on 5 µM formalin-fixed paraffin-embedded tissue sections. Heat-induced antigen retrieval (Tris-EDTA buffer at pH 9), blocking with 3% bovine serum albumin, and titrated antibody staining concentrations were employed. Slides were imaged using the Fluidigm Hyperion Tissue Imager system with a 1-µm^2^ laser ablation spot size at a frequency of 200 Hz and ablation energy of 3 dB. Area of analyzed tissue varied from 400 um^2^ up to 1000 um^2^ with multiple areas imaged per specimen. Image analysis was performed with MCD viewer (Fluidigm) and HistoCAT [48].

### Clinical samples, immunohistochemistry (IHC), and analysis

The protocol was approved by the Institutional Ethics Committee of West China Hospital of Stomatology (Chengdu, Sichuan, China, WCHSIRB-ST-2014-091). Human gingival specimens were collected from crown lengthening surgery (normal gingival tissues) and gingivectomy (periodontitis tissues) by informed consent.

Immunohistochemical localization of select proteins were performed on standard protocol on 4% paraformaldehyde fixed, paraffin embedded tissues using anti Luciferase (Novus Biologicals, Littleton, CO), NLRP3 (LifeSpan Biosciences), 8-OHdG (Abcam), TUNEL (Thermo Fisher Scientific Inc., Waltham, MA, USA), IL-1β (R&D Systems), Gr1.1 (Novus Biologicals), CD33 (Abcam), CD11b (Abcam), and Pan cytokeratin antibodies (Santa Cruz Biotechnology). Quantitation of positive staining was normalized to total number of cells per field by NIH Image software.

### Enzyme-linked immunosorbent assay (ELISA)

ELISA was performed for the expression of IL-1β (Biolegend, San Diego, CA), CCL2, CCL5, CXCL5 and CXCL12 (MultiSciences, Hangzhou, Zhejiang, China) on conditioned media from human gingival fibroblasts expanded for 24 h in the presence or absence of LPS. IL-1β ELISA was also performed on conditioned media from mouse gingival fibroblasts and blood plasma from mice of early metastasis models.

### Statistical analysis

Data were expressed as mean ± SEM. The statistical significance of differences was assessed by using ANOVA or student’s *t* test. Difference was considered significant when *P* < 0.05.

## Supplementary information

Supplement table 1

Supplement table 2

Supplemental Figure 1

Supplemental Figure 2

Supplemental Figure 3

## References

[1] Eke PI, Dye BA, Wei L, Slade GD, Thornton-Evans GO, Borgnakke WS (2015). Update on prevalence of periodontitis in adults in the United States: NHANES 2009 to 2012. J Periodontol.

[2] Han YW, Houcken W, Loos BG, Schenkein HA, Tezal M (2014). Periodontal disease, atherosclerosis, adverse pregnancy outcomes, and head-and-neck cancer. Adv Dent Res.

[3] Kruger M, Hansen T, Kasaj A, Moergel M (2013). The correlation between chronic periodontitis and oral cancer. Case Rep Dent.

[4] Zeng XT, Deng AP, Li C, Xia LY, Niu YM, Leng WD (2013). Periodontal disease and risk of head and neck cancer: a meta-analysis of observational studies. PLoS ONE.

[5] Yao QW, Zhou DS, Peng HJ, Ji P, Liu DS (2014). Association of periodontal disease with oral cancer: a meta-analysis. Tumour Biol.

[6] Moergel M, Kammerer P, Kasaj A, Armouti E, Alshihri A, Weyer V (2013). Chronic periodontitis and its possible association with oral squamous cell carcinoma—a retrospective case control study. Head Face Med.

[7] Nagy KN, Sonkodi I, Szoke I, Nagy E, Newman HN (1998). The microflora associated with human oral carcinomas. Oral Oncol.

[8] Correa P, Piazuelo MB (2011). Helicobacter pylori Infection and gastric adenocarcinoma. US Gastroenterol Hepatol Rev.

[9] Pendyala G, Joshi S, Chaudhari S, Gandhage D (2013). Links demystified: periodontitis and cancer. Dent Res J.

[10] Linden G, Patterson C, Evans A, Kee F (2007). Obesity and periodontitis in 60–70-year-old men. J Clin Periodontol.

[11] Kshirsagar AV, Moss KL, Elter JR, Beck JD, Offenbacher S, Falk RJ (2005). Periodontal disease is associated with renal insufficiency in the Atherosclerosis Risk In Communities (ARIC) study. Am J Kidney Dis.

[12] Gholizadeh P, Eslami H, Kafil HS (2017). Carcinogenesis mechanisms of Fusobacterium nucleatum. Biomed Pharmacother.

[13] Castellarin M, Warren RL, Freeman JD, Dreolini L, Krzywinski M, Strauss J (2012). Fusobacterium nucleatum infection is prevalent in human colorectal carcinoma. Genome Res.

[14] Coussens LM, Werb Z (2002). Inflammation and cancer. Nature.

[15] Cheng R, Liu W, Zhang R, Feng Y, Bhowmick NA, Hu T (2017). Porphyromonas gingivalis-derived lipopolysaccharide combines hypoxia to induce caspase-1 activation in periodontitis. Front Cell Infect Microbiol.

[16] Yokoyama M, Ukai T, Ayon Haro ER, Kishimoto T, Yoshinaga Y, Hara Y (2011). Membrane-bound CD40 ligand on T cells from mice injected with lipopolysaccharide accelerates lipopolysaccharide-induced osteoclastogenesis. J Periodontal Res.

[17] Bode KA, Schmitz F, Vargas L, Heeg K, Dalpke AH (2009). Kinetic of RelA activation controls magnitude of TLR-mediated IL-12p40 induction. J Immunol.

[18] Higgins SC, Lavelle EC, McCann C, Keogh B, McNeela E, Byrne P (2003). Toll-like receptor 4-mediated innate IL-10 activates antigen-specific regulatory T cells and confers resistance to Bordetella pertussis by inhibiting inflammatory pathology. J Immunol.

[19] Mora J, Weigert A (2016). IL-1 family cytokines in cancer immunity - a matter of life and death. Biol Chem.

[20] Garlanda C, Dinarello CA, Mantovani A (2013). The interleukin-1 family: back to the future. Immunity.

[21] Lukens JR, Gross JM, Kanneganti TD (2012). IL-1 family cytokines trigger sterile inflammatory disease. Front Immunol.

[22] Bar D, Apte RN, Voronov E, Dinarello CA, Cohen S (2004). A continuous delivery system of IL-1 receptor antagonist reduces angiogenesis and inhibits tumor development. FASEB J.

[23] Song X, Voronov E, Dvorkin T, Fima E, Cagnano E, Benharroch D (2003). Differential effects of IL-1 alpha and IL-1 beta on tumorigenicity patterns and invasiveness. J Immunol.

[24] Voronov E, Shouval DS, Krelin Y, Cagnano E, Benharroch D, Iwakura Y (2003). IL-1 is required for tumor invasiveness and angiogenesis. Proc Natl Acad Sci USA.

[25] Guo B, Fu S, Zhang J, Liu B, Li Z (2016). Targeting inflammasome/IL-1 pathways for cancer immunotherapy. Sci Rep.

[26] Condamine T, Ramachandran I, Youn JI, Gabrilovich DI (2015). Regulation of tumor metastasis by myeloid-derived suppressor cells. Annu Rev Med.

[27] Huang A, Zhang B, Wang B, Zhang F, Fan KX, Guo YJ (2013). Increased CD14(+)HLA-DR (−/low) myeloid-derived suppressor cells correlate with extrathoracic metastasis and poor response to chemotherapy in non-small cell lung cancer patients. Cancer Immunol Immunother.

[28] Yu J, Du W, Yan F, Wang Y, Li H, Cao S (2013). Myeloid-derived suppressor cells suppress antitumor immune responses through IDO expression and correlate with lymph node metastasis in patients with breast cancer. J Immunol.

[29] Achberger S, Aldrich W, Tubbs R, Crabb JW, Singh AD, Triozzi PL (2014). Circulating immune cell and microRNA in patients with uveal melanoma developing metastatic disease. Mol Immunol.

[30] Kaplan RN, Rafii S, Lyden D (2006). Preparing the “soil”: the premetastatic niche. Cancer Res.

[31] Su L, Xu Q, Zhang P, Michalek SM, Katz J (2017). Phenotype and function of myeloid-derived suppressor cells induced by porphyromonas gingivalis infection. Infect Immun..

[32] Ray A, Chakraborty K, Ray P (2013). Immunosuppressive MDSCs induced by TLR signaling during infection and role in resolution of inflammation. Front Cell Infect Microbiol.

[33] Abais JM, Xia M, Zhang Y, Boini KM, Li PL (2015). Redox regulation of NLRP3 inflammasomes: ROS as trigger or effector?. Antioxid Redox Signal.

[34] Kumar V, Patel S, Tcyganov E, Gabrilovich DI (2016). The nature of myeloid-derived suppressor cells in the tumor microenvironment. Trends Immunol.

[35] Sampson TR, Debelius JW, Thron T, Janssen S, Shastri GG, Ilhan ZE (2016). Gut microbiota regulate motor deficits and neuroinflammation in a model of Parkinson’s disease. Cell.

[36] Huang G, Xu J, Lefever DE, Glenn TC, Nagy T, Guo TL (2017). Genistein prevention of hyperglycemia and improvement of glucose tolerance in adult non-obese diabetic mice are associated with alterations of gut microbiome and immune homeostasis. Toxicol Appl Pharmacol.

[37] Schugar RC, Willard B, Wang Z, Brown JM (2018). Postprandial gut microbiota-driven choline metabolism links dietary cues to adipose tissue dysfunction. Adipocyte.

[38] Casella G, Garzetti L, Gatta AT, Finardi A, Maiorino C, Ruffini F (2016). IL4 induces IL6-producing M2 macrophages associated to inhibition of neuroinflammation in vitro and in vivo. J Neuroinflammation.

[39] Youn JI, Nagaraj S, Collazo M, Gabrilovich DI (2008). Subsets of myeloid-derived suppressor cells in tumor-bearing mice. J Immunol.

[40] Gabrilovich DI, Ostrand-Rosenberg S, Bronte V (2012). Coordinated regulation of myeloid cells by tumours. Nat Rev Immunol.

[41] Cheng R, Feng Y, Zhang R, Liu W, Lei L, Hu T (2018). The extent of pyroptosis varies in different stages of apical periodontitis. Biochim Biophys Acta.

[42] Gomes FI, Aragao MG, Barbosa FC, Bezerra MM, de Paulo Teixeira Pinto V, Chaves HV (2016). Inflammatory cytokines interleukin-1beta and tumour necrosis factor-alpha—novel biomarkers for the detection of periodontal diseases: a literature review. J Oral Maxillofac Res.

[43] Zhang Y, Lv D, Kim HJ, Kurt RA, Bu W, Li Y (2013). A novel role of hematopoietic CCL5 in promoting triple-negative mammary tumor progression by regulating generation of myeloid-derived suppressor cells. Cell Res.

[44] Haldar S, Dru C, Choudhury D, Mishra R, Fernandez A, Biondi S (2015). Inflammation and pyroptosis mediate muscle expansion in an interleukin-1beta (IL-1beta)-dependent manner. J Biol Chem.

[45] Haldar S, Dru C, Mishra R, Tripathi M, Duong F, Angara B (2016). Histone deacetylase inhibitors mediate DNA damage repair in ameliorating hemorrhagic cystitis. Sci Rep.

[46] Thi HTH, Hong S (2017). Inflammasome as a therapeutic target for cancer prevention and treatment. J Cancer Prev.

[47] Saijo Y, Tanaka M, Miki M, Usui K, Suzuki T, Maemondo M (2002). Proinflammatory cytokine IL-1 beta promotes tumor growth of Lewis lung carcinoma by induction of angiogenic factors: in vivo analysis of tumor-stromal interaction. J Immunol.

[48] Schapiro D, Jackson HW, Raghuraman S, Fischer JR, Zanotelli VRT, Schulz D (2017). histoCAT: analysis of cell phenotypes and interactions in multiplex image cytometry data. Nat Methods.

