# Supplementary material for: Periodontal inflammation recruits distant metastatic breast cancer cells by increasing myeloid-derived suppressor cells
Source: Oncogene. 2019 Nov 4;39(7):1543–56. doi: 10.1038/s41388-019-1084-z (PMC7018659; doi:10.1038/s41388-019-1084-z)
Supplement: Supplementary file 1 — Supplement table 1 [file 41388_2019_1084_MOESM1_ESM.pdf]

Supplement table 1

	Forward	Reverse
tlr2	5’-TGCTTTCCTGCTGGAGATTT-3’	5’-TGTAACGCAACAGCTTCAGG-3’
tlr4	5’-CTGATGACATTCCTTCTTCAAC-3’	5’-TTTCCTGTCAGTATCAAGTTTG-3’
il-1β	5’-ACCTAGCTGTCAACGTGTGG-3’	5’-TCAAAGCAATGTGCTGGTGC-3’
ccl2	5’-GCCCCACTCACCTGCTGCTACT-3’	5’-CCTGCTGCTGGTGATCCTCCTGT-3’
ccl5	5’-AGTCGTGTTTGTCACTCGAAGGA-3’	5’-AGTTGATGTATTCTTGAACCCACTTC-3’
cxcl5	5’-CGTAACTCCAAAAATTAATCCCAAA-3’	5’-CGAGTGCATTCCGCTTAGCT-3’
cxcl12	5’-GTGGGCCGCTCTAGGCACCA-3’	5’-CGGTTCGCCTTAGGGTTCAGG-3’
β-actin	5’-GTGGGCCGCTCTAGGCACCA-3’	5’-CGGTTGGCCTTAGGGTTCAGG-3’
