# Supplementary material for: Periodontal inflammation recruits distant metastatic breast cancer cells by increasing myeloid-derived suppressor cells
Source: Oncogene. 2019 Nov 4;39(7):1543–56. doi: 10.1038/s41388-019-1084-z (PMC7018659; doi:10.1038/s41388-019-1084-z)
Supplement: Supplementary file 2 — Supplement table 2 [file 41388_2019_1084_MOESM2_ESM.pdf]

Supplement table 2

No.	Subsets	Isotopes	Antibodies	Clones	Sources
1	Cytokine	141Pr	IL-10	JES5-16E3	BioLegend
2	Cytokine	152Sm	TGF-beta	1D11.16.8	Novus Biological
3	Cytokine	172Yb	IL-2	JES6-5H4	BioLegend
4	M2 Macrophages	155Gd	CD163	EPR19518	Abcam
5	MDSC	149Sm	CD11b	M170	BioLegend
6	MDSC	154Sm	CD11c	N418	BioLegend
8	MDSC	164Dy	Ly6C	HK1.4	BioLegend
9	MDSC	176Yb	Ly6G	1A8	BioLegend
10	M1 Macrophage	151Eu	MHC-II	M5/114.15.2	BioLegend
