# Supplementary material for: Periodontal inflammation recruits distant metastatic breast cancer cells by increasing myeloid-derived suppressor cells
Source: Oncogene. 2019 Nov 4;39(7):1543–56. doi: 10.1038/s41388-019-1084-z (PMC7018659; doi:10.1038/s41388-019-1084-z)
Supplement: Supplementary file 3 — Supplemental Figure 1 [file 41388_2019_1084_MOESM3_ESM.pdf]

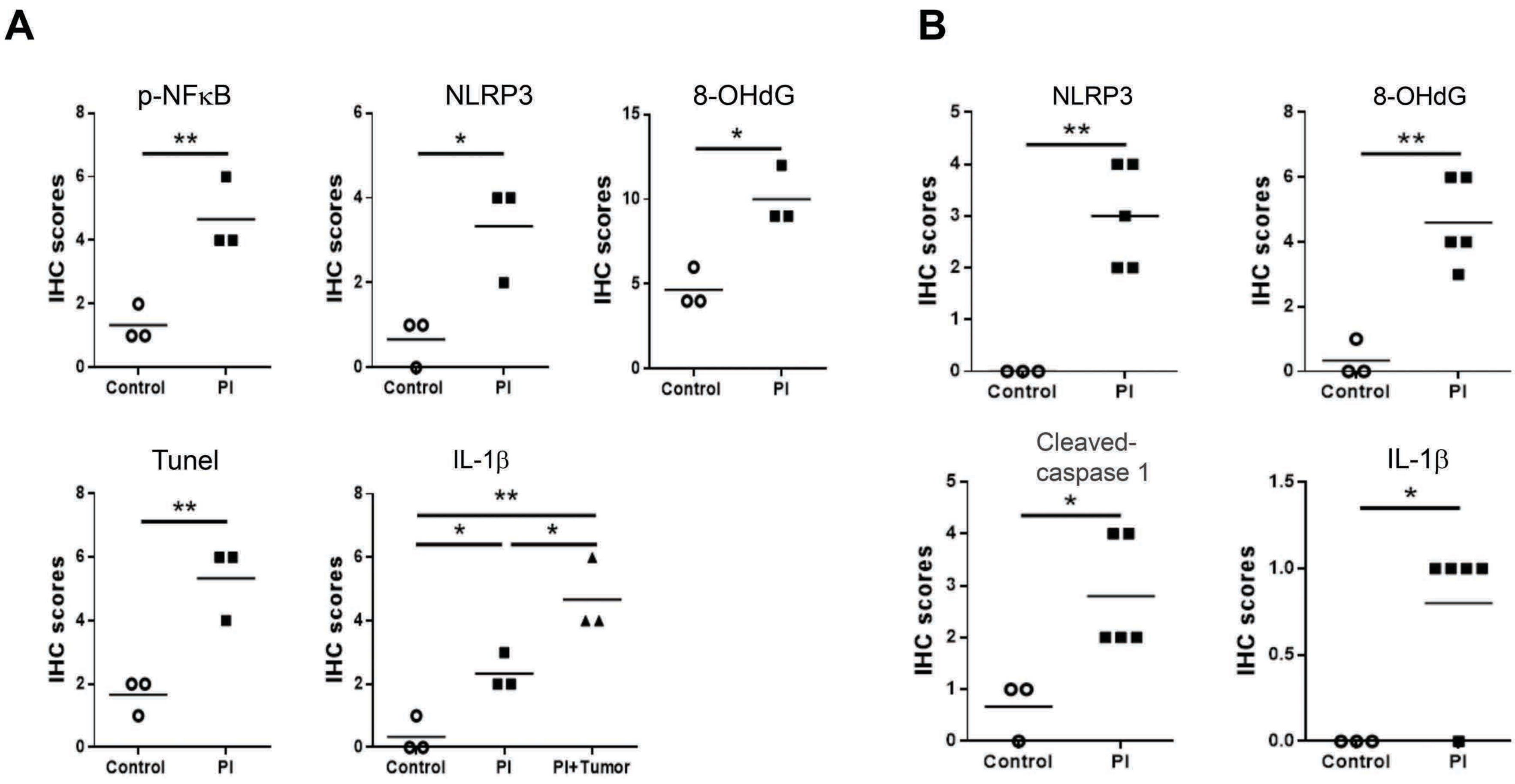

Figure S1. IHC Score of Figure 3 and Figure 5

IHC scoring was established according to standard protocol from the IHC of Fig 3A (A) and Fig 5A (B). Briefly, IHC score was evaluated from the multiplication between positive cells proportion score (0 for <5% of the cells with positive staining; 1 for 5 to 25 % of the cells with positive staining; 2, for 25 to 50 % of the cells with positive staining; 3, from 50 to 75 % of the cells with positive staining; 4, for more than 75% of the cells with staining) and staining intensity score (0, no; 1, weak ; 2, mild; 3, strong staining)."
