# Supplementary material for: Periodontal inflammation recruits distant metastatic breast cancer cells by increasing myeloid-derived suppressor cells
Source: Oncogene. 2019 Nov 4;39(7):1543–56. doi: 10.1038/s41388-019-1084-z (PMC7018659; doi:10.1038/s41388-019-1084-z)
Supplement: Supplementary file 4 — Supplemental Figure 2 [file 41388_2019_1084_MOESM4_ESM.pdf]

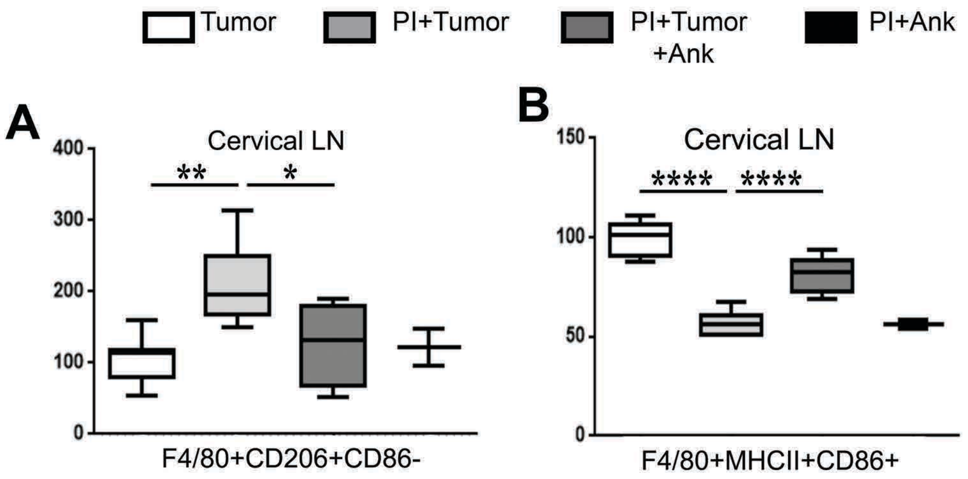

Figure S2. Evaluation of CD86, an M1 costimulatory molecule among macrophages.
(A) CD86+ expression was evaluated by FACS in the cervical LN. The levels of F4/80+CD206+CD86- M2 macrophages were significantly elevated in the cervical LN of PI + tumor animals and significantly reduced in the presence of Anakinra. (B) F4/80+MHCII+CD86+ M1 macrophages showed an opposite trend (student's t-test, **** $P<0.0001$).
