# Supplementary material for: Periodontal inflammation recruits distant metastatic breast cancer cells by increasing myeloid-derived suppressor cells
Source: Oncogene. 2019 Nov 4;39(7):1543–56. doi: 10.1038/s41388-019-1084-z (PMC7018659; doi:10.1038/s41388-019-1084-z)
Supplement: Supplementary file 5 — Supplemental Figure 3 [file 41388_2019_1084_MOESM5_ESM.pdf]

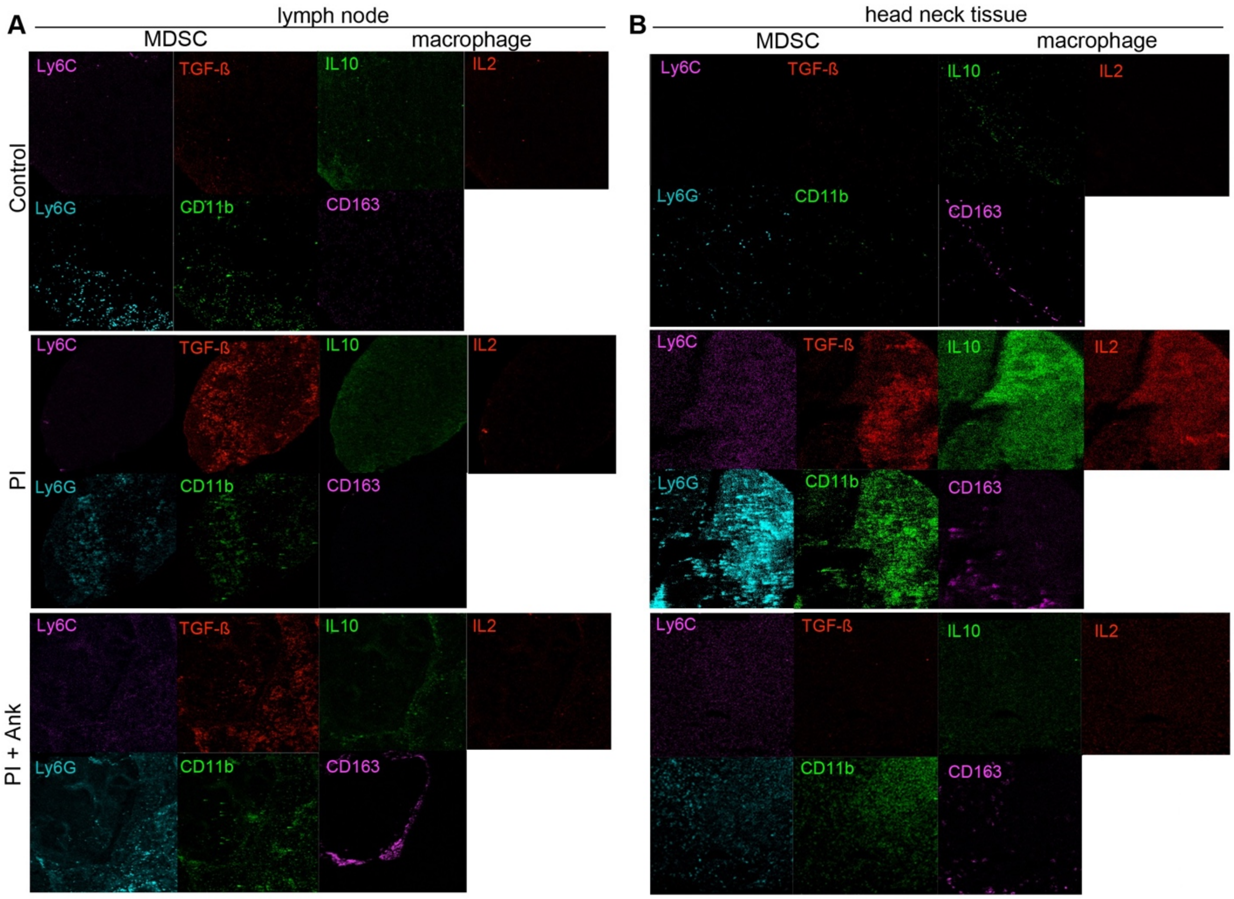

Supplemental Figure 3. Individual pseudo-colored panels for the indicated markers for MDSC, macrophage, and cytokines detected by CyTOF.
